# Norfloxacin sesquihydrate

**DOI:** 10.1107/S160053680900066X

**Published:** 2009-01-14

**Authors:** Nittala V. Ravindra, Gopal M. Panpalia, A. R. P. Sarma Jagarlapudi

**Affiliations:** a Birla Institute of Technology, Department of Pharmaceutical Sciences, Mesra, Ranchi 835 215, India; b GVK Biosciences Private Limited, S-1, Phase-1 Technocrats Industrial Estate, Balanagar, Hyderabad 500 037, India

## Abstract

In the crystal structure of the title compound [systematic name: 1-ethyl-6-fluoro-4-oxo-7-(piperazin-4-ium-1-yl)-1,4-dihydro­quinoline-3-carboxyl­ate sesquihydrate], C_16_H_18_FN_3_O_3_·1.42H_2_O, N—H⋯O and O—H⋯O hydrogen bonds assemble the mol­ecules in a two-dimensional layered corrugated sheet structure parallel to the *b* axis. The water mol­ecules are disordered [occupancies 0.741 (11) and 0.259 (11)].

## Related literature

For related structures, see: Yuasa *et al.* (1982 ▶); Windholz *et al.* (1983 ▶); Katdare *et al.* (1986 ▶); Šuštar *et al.* (1993 ▶); Florence *et al.* (2000 ▶); Barbas *et al.* (2006 ▶); Basavoju *et al.* (2006 ▶); Barbas *et al.* (2007 ▶); Chongcharoen *et al.* (2008 ▶)
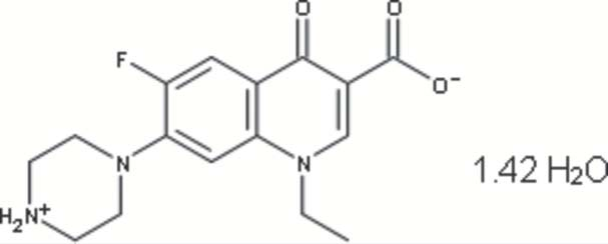


## Experimental

### 

#### Crystal data

C_16_H_18_FN_3_O_3_·1.42H_2_O
*M* *_r_* = 344.12
Monoclinic, 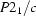

*a* = 8.8434 (18) Å
*b* = 22.312 (5) Å
*c* = 8.7564 (18) Å
β = 109.35 (3)°
*V* = 1630.2 (7) Å^3^
*Z* = 4
Mo *K*α radiation
μ = 0.11 mm^−1^
*T* = 298 (2) K
0.20 × 0.20 × 0.10 mm

#### Data collection

Bruker SMART CCD area-detector diffractometer
Absorption correction: none
16819 measured reflections
3228 independent reflections
2553 reflections with *I* > 2σ(*I*)
*R*_int_ = 0.044

#### Refinement

*R*[*F* ^2^ > 2σ(*F* ^2^)] = 0.081
*wR*(*F* ^2^) = 0.218
*S* = 1.03
3228 reflections
253 parameters
H atoms treated by a mixture of independent and constrained refinement
Δρ_max_ = 0.37 e Å^−3^
Δρ_min_ = −0.26 e Å^−3^

### 

Data collection: *SMART* (Bruker, 1997 ▶); cell refinement: *SAINT* (Bruker, 1997 ▶); data reduction: *SAINT*; program(s) used to solve structure: *SHELXS97* (Sheldrick, 2008 ▶); program(s) used to refine structure: *SHELXL97* (Sheldrick, 2008 ▶); molecular graphics: *SHELXTL* (Sheldrick, 2008 ▶); software used to prepare material for publication: *SHELXTL*.

## Supplementary Material

Crystal structure: contains datablocks I, global. DOI: 10.1107/S160053680900066X/gw2057sup1.cif

Structure factors: contains datablocks I. DOI: 10.1107/S160053680900066X/gw2057Isup2.hkl

Additional supplementary materials:  crystallographic information; 3D view; checkCIF report

## Figures and Tables

**Table 1:** Hydrogen-bond geometry (Å, °)

*D*—H⋯*A*	*D*—H	H⋯*A*	*D*⋯*A*	*D*—H⋯*A*
N3—H3*A*⋯O4*A*	0.90	1.88 (1)	2.741	160
N3—H3*A*⋯O4*B*	0.90	2.10 (1)	2.952	157
N3—H3*B*⋯O2^i^	0.90	1.99	2.777 (4)	145
N3—H3*B*⋯O3^i^	0.90	2.15	2.793 (4)	128
O4*B*—H4*B*⋯O1^ii^	0.912 (7)	2.02 (7)	2.793	141
O4*A*—H4*A*⋯O2^iii^	0.933 (10)	1.90 (9)	2.811	165

Symmetry codes: (i) 
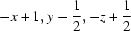
; (ii) 
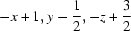
; (iii) 
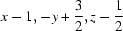
.

**Fig. 1.**
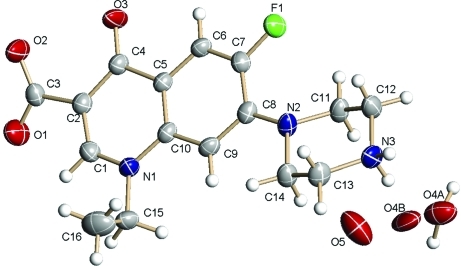
The molecular structure of (I), with atom labels and 50% probability displacement ellipsoids for non-H atoms. Labels for Hydrogen atoms have been omitted for clarity.

**Fig. 2.**
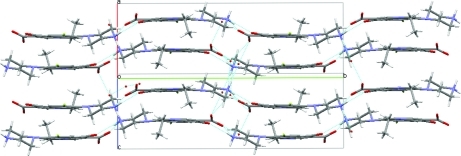
The packing of (I), showing the corrugated sheet layers of molecules parallel to b axis and water molecules connected by N—H···O and O—H···O hydrogen bonds (dashed lines).

## supplementary crystallographic information

### Comment

Norfloxacin (NF) is a broad spectrum 4-fluoroquinolone antibacterial used in the treatment of urinary tract infections. As part of our interest in polymorphs and hydrates of NF, we have investigated the crystal structure of NF sesquihydrate, (I) (Fig. 1). NF molecule is zwitterionic and the N3 nitrogen is protonated similar to the reported structure of dihydrate (Florence *et al*., 2000) and anhydrous zwitterion (Barbas *et al.*, 2007). The molecules are linked *via* N—H···O and O—H···O hydrogen bonds, forming two-dimensional corrugated sheets parallel to *b* axis. These sheets are linked together by the water molecules which act as acceptors of H atoms, assembling the molecules in an infinite two-dimensional network (Fig. 2).

### Experimental

The title compound was prepared from anhydrous form as reported by Katdare *et al.*,(1986). It was then dissolved in Acetonitrile on water bath and allowed to cool in sealed flask. Pale yellow colored block like crystals suitable for *x*-ray analysis appeared after two days.

### Refinement

The lattice water molecules are disordered. The O4 oxygen is disordered over two sites, O4A and O4B, with occupancies of 0.741 and 0.259 respectively. The O5 oxygen atom has a occupancy of 0.423. Due to disorder the hydrogen atoms on O5 oxygen could not be located. All H atoms were located from difference Fourier synthesis. Those bonded to O atoms were then refined independently and isotropically, whilst those attached to C and N atoms were placed in geometrically calculated positions and allowed to ride on their parent atoms with *U*_iso_(H) = 1.2Ueq(C), N—H = 0.90 Å, C—H distance restraints of 0.93, 0.96 and 0.97 Å for aromatic, methylene and methyl groups, respectively.

### Crystal data

**Table:**

C_16_H_18_FN_3_O_3_·1.42H_2_O	*F*(000) = 725.5
*M**_r_* = 344.12	*D*_x_ = 1.402 Mg m^−^^3^
Monoclinic, *P*2_1_/*c*	Melting point: 492.5(3) K
Hall symbol: -P 2ybc	Mo *K*α radiation, λ = 0.71073 Å
*a* = 8.8434 (18) Å	Cell parameters from 3228 reflections
*b* = 22.312 (5) Å	θ = 1.8–26.1°
*c* = 8.7564 (18) Å	µ = 0.11 mm^−^^1^
β = 109.35 (3)°	*T* = 298 K
*V* = 1630.2 (7) Å^3^	Block, pale yellow
*Z* = 4	0.20 × 0.20 × 0.10 mm

### Data collection

**Table:**

Bruker SMART CCD area-detector diffractometer	2553 reflections with *I* > 2σ(*I*)
Radiation source: fine-focus sealed tube	*R*_int_ = 0.044
graphite	θ_max_ = 26.1°, θ_min_ = 1.8°
φ and ω scans	*h* = −10→10
16819 measured reflections	*k* = −27→27
3228 independent reflections	*l* = −10→10

### Refinement

**Table:**

Refinement on *F*^2^	Primary atom site location: structure-invariant direct methods
Least-squares matrix: full	Secondary atom site location: difference Fourier map
*R*[*F*^2^ > 2σ(*F*^2^)] = 0.081	Hydrogen site location: inferred from neighbouring sites
*wR*(*F*^2^) = 0.218	H atoms treated by a mixture of independent and constrained refinement
*S* = 1.03	*w* = 1/[σ^2^(*F*_o_^2^) + (0.1124*P*)^2^ + 1.8021*P*] where *P* = (*F*_o_^2^ + 2*F*_c_^2^)/3
3228 reflections	(Δ/σ)_max_ < 0.001
253 parameters	Δρ_max_ = 0.37 e Å^−^^3^
0 restraints	Δρ_min_ = −0.26 e Å^−^^3^

### Special details

**Table:**

Geometry. All e.s.d.'s (except the e.s.d. in the dihedral angle between two l.s. planes) are estimated using the full covariance matrix. The cell e.s.d.'s are taken into account individually in the estimation of e.s.d.'s in distances, angles and torsion angles; correlations between e.s.d.'s in cell parameters are only used when they are defined by crystal symmetry. An approximate (isotropic) treatment of cell e.s.d.'s is used for estimating e.s.d.'s involving l.s. planes.
Refinement. Refinement of *F*^2^ against ALL reflections. The weighted *R*-factor *wR* and goodness of fit *S* are based on *F*^2^, conventional *R*-factors *R* are based on *F*, with *F* set to zero for negative *F*^2^. The threshold expression of *F*^2^ > σ(*F*^2^) is used only for calculating *R*-factors(gt) *etc*. and is not relevant to the choice of reflections for refinement. *R*-factors based on *F*^2^ are statistically about twice as large as those based on *F*, and *R*- factors based on ALL data will be even larger.

### Fractional atomic coordinates and isotropic or equivalent isotropic displacement parameters (Å^2^)

**Table:**

	*x*	*y*	*z*	*U*_iso_*/*U*_eq_	Occ. (<1)
F1	0.2851 (2)	0.73405 (8)	0.1027 (2)	0.0442 (5)	
C9	0.5896 (4)	0.69034 (13)	0.4775 (4)	0.0313 (7)	
H9	0.6340	0.6563	0.5365	0.038*	
N1	0.7787 (3)	0.75248 (11)	0.6815 (3)	0.0313 (6)	
C8	0.4641 (3)	0.68388 (13)	0.3326 (4)	0.0302 (7)	
C10	0.6513 (4)	0.74655 (12)	0.5373 (4)	0.0282 (6)	
N2	0.4098 (3)	0.62818 (11)	0.2644 (3)	0.0338 (6)	
C6	0.4554 (4)	0.79261 (13)	0.3075 (4)	0.0304 (7)	
H6	0.4079	0.8265	0.2498	0.037*	
C7	0.4011 (4)	0.73759 (13)	0.2506 (4)	0.0312 (7)	
C4	0.6462 (4)	0.85899 (13)	0.5062 (4)	0.0313 (7)	
C1	0.8307 (4)	0.80766 (13)	0.7354 (4)	0.0327 (7)	
H1	0.9128	0.8101	0.8344	0.039*	
C2	0.7747 (4)	0.86019 (13)	0.6590 (4)	0.0315 (7)	
O1	0.9214 (3)	0.91437 (11)	0.8908 (3)	0.0543 (7)	
O3	0.5884 (3)	0.90336 (10)	0.4227 (3)	0.0509 (7)	
O2	0.8443 (3)	0.96306 (11)	0.6594 (3)	0.0563 (8)	
C5	0.5828 (3)	0.79879 (12)	0.4528 (3)	0.0278 (6)	
C3	0.8524 (4)	0.91709 (13)	0.7418 (4)	0.0330 (7)	
C14	0.4966 (4)	0.57470 (13)	0.3422 (4)	0.0378 (8)	
H14A	0.4622	0.5633	0.4325	0.045*	
H14B	0.6105	0.5832	0.3838	0.045*	
N3	0.2914 (3)	0.51183 (11)	0.1476 (3)	0.0382 (7)	
H3A	0.2528	0.4972	0.2232	0.046*	
H3B	0.2762	0.4840	0.0697	0.046*	
C15	0.8690 (4)	0.70072 (14)	0.7748 (4)	0.0391 (8)	
H15A	0.9077	0.7109	0.8890	0.047*	
H15B	0.7972	0.6667	0.7607	0.047*	
C12	0.2026 (4)	0.56722 (14)	0.0767 (4)	0.0427 (8)	
H12A	0.0884	0.5589	0.0367	0.051*	
H12B	0.2344	0.5803	−0.0138	0.051*	
C11	0.2368 (4)	0.61614 (14)	0.2023 (4)	0.0381 (8)	
H11A	0.1796	0.6523	0.1545	0.046*	
H11B	0.2002	0.6039	0.2904	0.046*	
C13	0.4652 (4)	0.52396 (14)	0.2223 (4)	0.0393 (8)	
H13A	0.5099	0.5339	0.1381	0.047*	
H13B	0.5186	0.4881	0.2767	0.047*	
C16	1.0069 (5)	0.6835 (2)	0.7236 (5)	0.0635 (12)	
H16A	0.9684	0.6701	0.6132	0.095*	
H16B	1.0656	0.6517	0.7916	0.095*	
H16C	1.0758	0.7175	0.7327	0.095*	
O4A	0.1091 (6)	0.4638 (2)	0.3166 (6)	0.0616 (18)	0.741 (11)
O5	0.2349 (14)	0.5705 (7)	0.6166 (16)	0.076 (3)	0.423 (6)
O4B	0.1742 (14)	0.4991 (6)	0.4249 (17)	0.059 (5)	0.259 (11)
H4B	0.134 (8)	0.463 (3)	0.440 (8)	0.13 (2)*	
H4A	0.020 (11)	0.489 (4)	0.282 (10)	0.19 (4)*	

### Atomic displacement parameters (Å^2^)

**Table:**

	*U*^11^	*U*^22^	*U*^33^	*U*^12^	*U*^13^	*U*^23^
F1	0.0476 (11)	0.0359 (10)	0.0394 (11)	−0.0060 (8)	0.0016 (9)	−0.0039 (8)
C9	0.0353 (16)	0.0209 (14)	0.0396 (17)	0.0036 (12)	0.0149 (13)	0.0037 (12)
N1	0.0330 (14)	0.0235 (12)	0.0355 (14)	0.0007 (10)	0.0086 (11)	0.0014 (10)
C8	0.0312 (15)	0.0244 (15)	0.0392 (17)	−0.0002 (12)	0.0170 (13)	−0.0039 (12)
C10	0.0320 (15)	0.0221 (14)	0.0342 (16)	−0.0016 (11)	0.0160 (13)	−0.0018 (12)
N2	0.0301 (14)	0.0221 (13)	0.0485 (16)	−0.0002 (10)	0.0118 (12)	−0.0066 (11)
C6	0.0339 (16)	0.0232 (14)	0.0351 (16)	0.0020 (12)	0.0128 (13)	0.0033 (12)
C7	0.0298 (15)	0.0320 (16)	0.0313 (16)	−0.0018 (12)	0.0094 (12)	−0.0039 (12)
C4	0.0355 (16)	0.0229 (14)	0.0350 (16)	−0.0020 (12)	0.0112 (13)	−0.0017 (12)
C1	0.0321 (16)	0.0328 (16)	0.0314 (16)	0.0006 (13)	0.0082 (12)	−0.0010 (13)
C2	0.0330 (16)	0.0275 (15)	0.0348 (16)	−0.0018 (12)	0.0123 (13)	−0.0026 (12)
O1	0.0660 (17)	0.0436 (15)	0.0409 (14)	−0.0094 (12)	0.0010 (12)	−0.0095 (11)
O3	0.0640 (17)	0.0217 (11)	0.0489 (15)	−0.0043 (11)	−0.0056 (12)	0.0063 (10)
O2	0.0724 (18)	0.0306 (13)	0.0495 (15)	−0.0186 (12)	−0.0017 (13)	0.0018 (11)
C5	0.0313 (15)	0.0228 (14)	0.0314 (15)	−0.0014 (11)	0.0134 (12)	−0.0009 (11)
C3	0.0289 (15)	0.0274 (16)	0.0406 (18)	0.0006 (12)	0.0086 (13)	−0.0054 (13)
C14	0.0335 (16)	0.0258 (15)	0.051 (2)	0.0005 (13)	0.0106 (14)	−0.0029 (14)
N3	0.0458 (16)	0.0211 (13)	0.0436 (16)	−0.0045 (11)	0.0093 (13)	−0.0048 (11)
C15	0.0448 (19)	0.0286 (17)	0.0395 (18)	0.0020 (14)	0.0080 (14)	0.0079 (13)
C12	0.0379 (18)	0.0264 (16)	0.054 (2)	−0.0032 (13)	0.0015 (15)	−0.0004 (14)
C11	0.0303 (17)	0.0266 (16)	0.056 (2)	−0.0009 (12)	0.0121 (14)	−0.0022 (14)
C13	0.0409 (18)	0.0226 (15)	0.054 (2)	0.0026 (13)	0.0153 (15)	−0.0055 (14)
C16	0.060 (3)	0.063 (3)	0.072 (3)	0.025 (2)	0.029 (2)	0.021 (2)
O4A	0.061 (3)	0.058 (3)	0.071 (3)	0.020 (2)	0.031 (2)	0.024 (3)
O5	0.048 (6)	0.101 (10)	0.066 (8)	0.015 (6)	0.003 (5)	−0.015 (7)
O4B	0.052 (7)	0.060 (9)	0.071 (10)	0.007 (6)	0.030 (6)	0.038 (8)

### Geometric parameters (Å, °)

**Table:**

F1—C7	1.361 (3)	C14—C13	1.506 (4)
C9—C8	1.389 (4)	C14—H14A	0.9700
C9—C10	1.398 (4)	C14—H14B	0.9700
C9—H9	0.9300	N3—C13	1.483 (4)
N1—C1	1.344 (4)	N3—C12	1.485 (4)
N1—C10	1.392 (4)	N3—H3A	0.9000
N1—C15	1.486 (4)	N3—H3B	0.9000
C8—N2	1.393 (4)	C15—C16	1.483 (5)
C8—C7	1.413 (4)	C15—H15A	0.9700
C10—C5	1.406 (4)	C15—H15B	0.9700
N2—C14	1.459 (4)	C12—C11	1.507 (5)
N2—C11	1.469 (4)	C12—H12A	0.9700
C6—C7	1.352 (4)	C12—H12B	0.9700
C6—C5	1.399 (4)	C11—H11A	0.9700
C6—H6	0.9300	C11—H11B	0.9700
C4—O3	1.236 (4)	C13—H13A	0.9700
C4—C2	1.441 (4)	C13—H13B	0.9700
C4—C5	1.471 (4)	C16—H16A	0.9600
C1—C2	1.359 (4)	C16—H16B	0.9600
C1—H1	0.9300	C16—H16C	0.9600
C2—C3	1.510 (4)	O4A—H4B	1.03 (7)
O1—C3	1.245 (4)	O4A—H4A	0.93 (10)
O2—C3	1.242 (4)	O4B—H4B	0.91 (7)
			
C8—C9—C10	122.0 (3)	C13—C14—H14B	109.7
C8—C9—H9	119.0	H14A—C14—H14B	108.2
C10—C9—H9	119.0	C13—N3—C12	111.1 (2)
C1—N1—C10	119.0 (2)	C13—N3—H3A	109.4
C1—N1—C15	117.4 (3)	C12—N3—H3A	109.4
C10—N1—C15	123.4 (2)	C13—N3—H3B	109.4
C9—C8—N2	122.8 (3)	C12—N3—H3B	109.4
C9—C8—C7	115.9 (3)	H3A—N3—H3B	108.0
N2—C8—C7	121.1 (3)	C16—C15—N1	112.4 (3)
N1—C10—C9	121.6 (3)	C16—C15—H15A	109.1
N1—C10—C5	118.5 (2)	N1—C15—H15A	109.1
C9—C10—C5	120.0 (3)	C16—C15—H15B	109.1
C8—N2—C14	118.4 (3)	N1—C15—H15B	109.1
C8—N2—C11	119.4 (2)	H15A—C15—H15B	107.9
C14—N2—C11	110.2 (2)	N3—C12—C11	110.3 (3)
C7—C6—C5	120.4 (3)	N3—C12—H12A	109.6
C7—C6—H6	119.8	C11—C12—H12A	109.6
C5—C6—H6	119.8	N3—C12—H12B	109.6
C6—C7—F1	117.9 (3)	C11—C12—H12B	109.6
C6—C7—C8	123.4 (3)	H12A—C12—H12B	108.1
F1—C7—C8	118.7 (3)	N2—C11—C12	109.6 (3)
O3—C4—C2	125.4 (3)	N2—C11—H11A	109.7
O3—C4—C5	120.3 (3)	C12—C11—H11A	109.7
C2—C4—C5	114.4 (3)	N2—C11—H11B	109.7
N1—C1—C2	126.2 (3)	C12—C11—H11B	109.7
N1—C1—H1	116.9	H11A—C11—H11B	108.2
C2—C1—H1	116.9	N3—C13—C14	111.6 (3)
C1—C2—C4	119.2 (3)	N3—C13—H13A	109.3
C1—C2—C3	117.1 (3)	C14—C13—H13A	109.3
C4—C2—C3	123.7 (3)	N3—C13—H13B	109.3
C6—C5—C10	118.3 (3)	C14—C13—H13B	109.3
C6—C5—C4	119.1 (3)	H13A—C13—H13B	108.0
C10—C5—C4	122.5 (3)	C15—C16—H16A	109.5
O2—C3—O1	124.3 (3)	C15—C16—H16B	109.5
O2—C3—C2	119.1 (3)	H16A—C16—H16B	109.5
O1—C3—C2	116.7 (3)	C15—C16—H16C	109.5
N2—C14—C13	110.0 (3)	H16A—C16—H16C	109.5
N2—C14—H14A	109.7	H16B—C16—H16C	109.5
C13—C14—H14A	109.7	H4B—O4A—H4A	102 (6)
N2—C14—H14B	109.7		
			
C10—C9—C8—N2	174.5 (3)	C5—C4—C2—C3	−176.7 (3)
C10—C9—C8—C7	−0.8 (4)	C7—C6—C5—C10	−0.3 (4)
C1—N1—C10—C9	−178.4 (3)	C7—C6—C5—C4	176.0 (3)
C15—N1—C10—C9	6.1 (4)	N1—C10—C5—C6	179.7 (3)
C1—N1—C10—C5	0.5 (4)	C9—C10—C5—C6	−1.4 (4)
C15—N1—C10—C5	−175.0 (3)	N1—C10—C5—C4	3.5 (4)
C8—C9—C10—N1	−179.1 (3)	C9—C10—C5—C4	−177.6 (3)
C8—C9—C10—C5	2.0 (4)	O3—C4—C5—C6	−0.7 (4)
C9—C8—N2—C14	−4.8 (4)	C2—C4—C5—C6	178.6 (3)
C7—C8—N2—C14	170.3 (3)	O3—C4—C5—C10	175.5 (3)
C9—C8—N2—C11	134.2 (3)	C2—C4—C5—C10	−5.2 (4)
C7—C8—N2—C11	−50.7 (4)	C1—C2—C3—O2	157.3 (3)
C5—C6—C7—F1	−175.1 (3)	C4—C2—C3—O2	−22.9 (5)
C5—C6—C7—C8	1.6 (5)	C1—C2—C3—O1	−22.6 (4)
C9—C8—C7—C6	−1.0 (4)	C4—C2—C3—O1	157.2 (3)
N2—C8—C7—C6	−176.4 (3)	C8—N2—C14—C13	−157.6 (3)
C9—C8—C7—F1	175.7 (3)	C11—N2—C14—C13	59.9 (3)
N2—C8—C7—F1	0.3 (4)	C1—N1—C15—C16	−86.4 (4)
C10—N1—C1—C2	−2.7 (5)	C10—N1—C15—C16	89.2 (4)
C15—N1—C1—C2	173.1 (3)	C13—N3—C12—C11	−54.6 (4)
N1—C1—C2—C4	0.7 (5)	C8—N2—C11—C12	156.3 (3)
N1—C1—C2—C3	−179.5 (3)	C14—N2—C11—C12	−61.6 (3)
O3—C4—C2—C1	−177.6 (3)	N3—C12—C11—N2	58.6 (4)
C5—C4—C2—C1	3.1 (4)	C12—N3—C13—C14	53.4 (4)
O3—C4—C2—C3	2.5 (5)	N2—C14—C13—N3	−55.8 (4)

### Hydrogen-bond geometry (Å, °)

**Table:**

*D*—H···*A*	*D*—H	H···*A*	*D*···*A*	*D*—H···*A*
N3—H3A···O4A	0.90	1.88 (1)	2.741	160
N3—H3A···O4B	0.90	2.10 (1)	2.952	157
N3—H3B···O2^i^	0.90	1.99	2.777 (4)	145
N3—H3B···O3^i^	0.90	2.15	2.793 (4)	128
O4B—H4B···O1^ii^	0.912 (7)	2.02 (7)	2.793	141
O4A—H4A···O2^iii^	0.933 (10)	1.90 (9)	2.811	165

Symmetry codes: (i) −*x*+1, *y*−1/2, −*z*+1/2; (ii) −*x*+1, *y*−1/2, −*z*+3/2; (iii) *x*−1, −*y*+3/2, *z*−1/2.
